# Dynamic changes in vascular size and density in transgenic mice with Alzheimer’s disease

**DOI:** 10.18632/aging.103672

**Published:** 2020-09-09

**Authors:** Xiaowen Xu, Tong Meng, Qingqing Wen, Mengling Tao, Peijun Wang, Kai Zhong, Yong Shen

**Affiliations:** 1 Institute on Aging and Brain Disorders, First University Affiliated Hospital, Neurodegenerative Disorder Research Center, Division of Life and Medical Sciences, University of Science and Technology of China, Hefei Material Science National Laboratory at Microscale, CAS-Key Laboratory of Brain Functions and Brain Disorders, Center for Excellent in Brain Science and Intelligence Technology, Hefei, China; 2 Department of Radiology, Tongji Hospital, School of Medicine, Tongji University, Shanghai, China; 3 School of Medicine, Tongji University, Shanghai, China; 4 Key Laboratory for Biomedical Engineering of Ministry of Education, Department of Biomedical Engineering, College of Biomedical Engineering and Instrument Science, Zhejiang University, Hangzhou, Zhejiang, China; 5 High Magnetic Field Laboratory, Chinese Academy of Sciences, Hefei, China; 6 Key Laboratory of Anhui Province for High Field Magnetic Resonance Imaging, Hefei, China; 7 Center for Excellence in Brain Science and Intelligence Technology, Chinese Academy of Sciences, Shanghai, China

**Keywords:** Alzheimer's disease, hippocampus, magnetic resonance imaging, vessel size imaging, early diagnosis

## Abstract

Alzheimer's disease (AD) is one of the most common neurodegenerative diseases. Here, we used vessel size imaging to investigate the specific microvascular changes and most susceptible brain regions during AD progression in an amyloid precursor protein 23 (APP23) transgenic AD mouse model. Using 9.4 Tesla magnetic resonance imaging (MRI), the values of microvascular density (Density), mean vessel diameter (mVD), and vessel size index (VSI) were compared between APP23 and wild-type (WT) mice at 3, 6, 9, 14, and 20 months of age. Our results demonstrate that in 20-month old APP23 and WT mice, the Density values were significantly decreased, while the vascular dilatation and diameter had increased. However, a transient increase in the cortex Density at 14-months was observed in APP23 mice. Additionally, our results suggest that the hippocampus is the susceptible brain region affected by the abnormal microvascular angiogenesis during the early stages of AD. Together, our findings indicate that vessel size imaging using MRI can provide novel biomarkers for the early detection of AD, and for monitoring the effects of vascular-targeted therapeutics in AD.

## INTRODUCTION

Alzheimer's disease (AD) is a progressive neurodegenerative disease characterized by beta amyloid (Aβ) plaque deposition, neurofibrillary tangles, neuroinflammatory responses, synaptic degeneration, and extensive neuron loss [1–3]. Cerebrovascular abnormalities, such as blood brain barrier (BBB) dysfunction, endothelial injury, and hypoperfusion often occur during AD pathogenesis [4–6]. However, the specific changes in cerebrovascular density and morphology in AD are still unclear.

Vessel size imaging is a quantitative imaging method, which can evaluate the vascular angiogenesis and morphology by determining the parameters of microvascular density (Density), mean vessel diameter in the voxel (mVD), and vessel size index (VSI) [7, 8]. The parameters of Density and mVD represent the density and size of microvessels in the local vascular network, respectively. The VSI, a quantitative index, can monitor expansion and contraction of microvessels in vivo by revealing the distribution of microvascular diameters within the voxel [7, 9, 10]. These three parameters obtained by vessel size imaging have been used to explore the vascular angiogenesis and morphology of tumors and strokes [8, 10–12], but so far, no study has applied this method to evaluate the changes in cerebral microvessels in AD.

Given the robust association of cerebrovascular pathology with AD, and the paucity of non-invasive early-stage AD diagnostics, we investigated brain cerebrovascular density and morphology changes in an amyloid precursor protein 23 (APP23) transgenic mouse AD model by vessel size imaging using a 9.4 Tesla magnetic resonance machine [13, 14]. Our results demonstrate that the late stages of AD progression in APP23 mice are associated with the decreased Density of cerebral microvessels, increased vascular dilatation, and vascular diameter enlargement. In addition, our data show that abnormal vascular density and morphology changes associated with AD can be identified as early as 9 months in the AD mouse model, and that hippocampus is the sensitive region that reflects the microvessel changes in AD.

## RESULTS

### Density values in cortex and hippocampus of APP23 and WT mice

**Figure 1:**
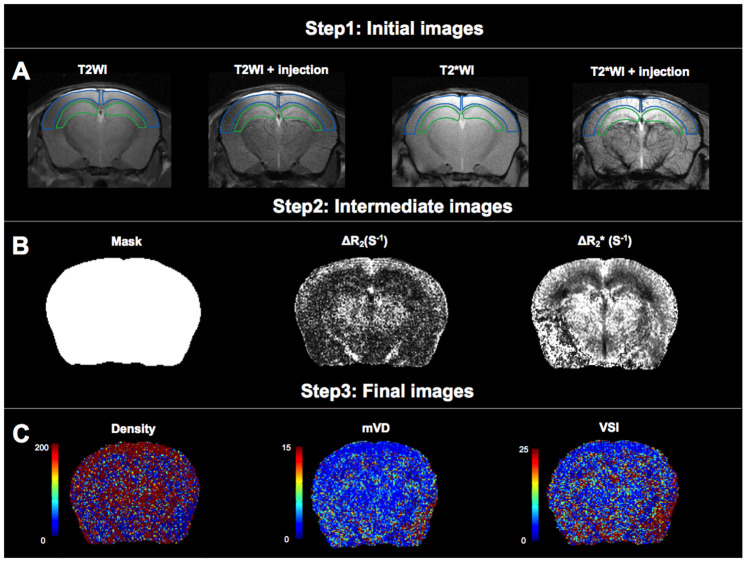
**Analysis of microvascular density (Density), mean vessel diameter in the voxel (mVD), and vessel size index (VSI).** (**A**) The first step of data processing. Two regions of interest (ROIs) were delineated on T_2_WI and projected to other images. The ROI delineated by the blue line is cortical region, and the ROI delineated by the green line is hippocampal region. (**B**) The second step of data processing. Maps of mask, ΔR_2_ and $\Delta {\text{R}}_{2}^{*}$ obtained from intermediate steps of data processing. (**C**) The third step of data processing. Colormaps of Density, mVD and VSI of obtained images.

Brain images of 46 mice were analyzed, including 24 WT mice and 22 APP23 mice, which have increased expression of the human amyloid precursor protein (APP). Processing of the obtained brain images is illustrated in Figure 1.

**Figure 2:**
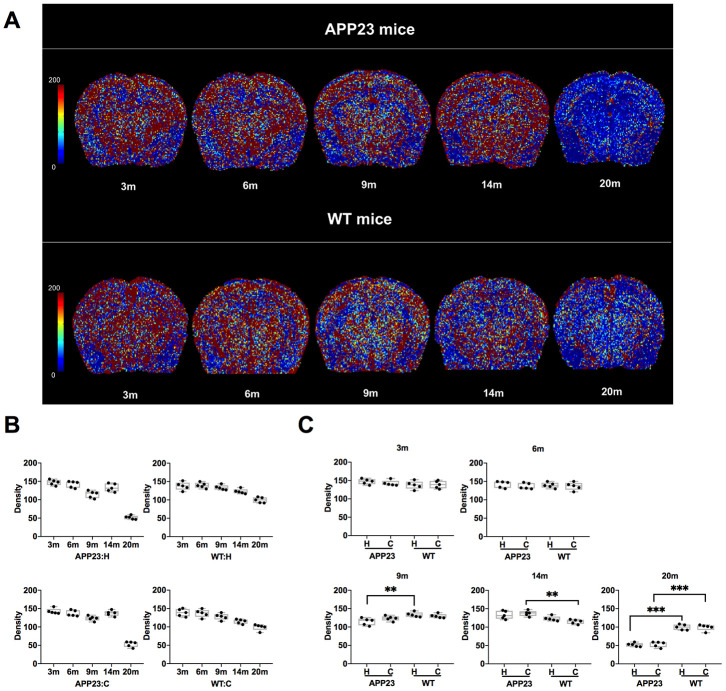
**Density in APP23 and WT mice of different ages.** (**A**) Colormap of Density in APP23 and WT mice of different ages. (**B**) Density values of cortex and hippocampus of APP23 and WT mice of different ages. (**C**) Comparison of the Density values in the cortex and hippocampus between APP23 and WT mice of the same age. WT: wide type; C: cortex; H: hippocampus; VSI, vessel size index.

The Density values of cortex and hippocampus in APP23 and WT mice of different ages are shown in Tables 1, 2 and in Figure 2. The colormap of Density in APP23 and WT mice at different ages was presented in Figure 2A. The Density values of both cortex and hippocampus in APP23 and WT mice decreased between 3 and 9 months (Figure 2B). However, the Density values of cortex and hippocampus differed between 14 month-old APP23 and WT mice. Compared with the gradual decrease of the Density in WT mice, the Density values of cortex and hippocampus in 14-month old APP23 mice showed a transient increase (Figure 2B). At 20 months, the Density values of cortical and hippocampal regions of APP23 and WT mice were significantly decreased. Specifically, the Density values of cortex and hippocampus in 20-month old APP23 mice were 63% and 65% lower than the highest values at 3 months, while they were only 29% and 28% lower in 20-month old WT mice than in 3-month WT mice. Therefore, at 20 months, the decrease in Density values of cortex and hippocampus was more obvious in APP23 mice than in WT mice.

**Table 1:** Density, mVD, and VSI values of cortex and hippocampus in APP23 transgenic mice.

**Age (months)**	**Cortical Region**				**Hippocampal Region**		
	**Density (vessel mm^-2^)**	**mVD (a.u)**	**VSI (μm)**		**Density (vessel mm^-2^)**	**mVD (a.u)**	**VSI (μm)**
**3**	142.58±8.66	6.90±0.72	10.70±1.52		147.10±8.71	6.38±0.41	10.31±1.52
**6**	137.13±7.23	6.67±0.58	10.32±1.22		142.04±8.04	6.33±0.63	10.01±0.88
**9**	123.52±5.98	6.57±0.67	9.28±0.94		114.62±7.73	6.29±0.39	8.32±0.91
**14**	137.27±9.77	5.56±0.83	8.09±1.19		133.09±9.85	6.21±0.52	7.40±0.90
**20**	53.15±6.76	13.41±1.23	21.00±1.28		51.78±4.65	13.36±0.85	22.16±2.65

mVD, mean vessel diameter in the voxel; VSI, vessel size index.

**Table 2:** Density, mVD, and VSI values of cortex and hippocampus of WT mice.

**Age (months)**	**Cortical Region**				**Hippocampal Region**		
	**Density (vessel mm^-2^)**	**mVD (a.u)**	**VSI (μm)**		**Density (vessel mm^-2^)**	**mVD (a.u)**	**VSI (μm)**
**3**	138.85±9.16	6.55±0.83	10.31±0.84		137.60±13.93	6.61±0.35	9.16±1.01
**6**	136.94±10.37	6.27±0.54	10.51±1.22		139.51±8.76	6.58±0.83	9.33±1.35
**9**	129.82±7.09	6.98±0.76	10.69±1.04		133.47±6.97	6.68±0.52	11.12±1.08
**14**	114.78±5.42	6.93±0.45	9.55±1.32		123.05±5.84	6.60±0.61	8.74±0.94
**20**	99.10±7.47	6.82±0.62	13.65±0.96		99.63±9.75	7.91±0.96	13.07±1.51

mVD, mean vessel diameter in the voxel; VSI, vessel size index.

The comparison of the Density values in cortex and hippocampus between APP23 and WT mice of the same age is shown in Figure 2C. In the cortical area, the Density value of APP23 mice was significantly higher than that in WT mice at 14 months. At 20 months, the Density value of cortex in APP23 mice was significantly lower than in WT mice (Figure 2C). However, in the hippocampal area, the Density value in APP23 mice was significantly lower than in WT mice at 9 months, and was further reduced at 20 months (Figure 2C). Therefore, the Density values suggest that significant differences in the hippocampus between APP23 and WT mice are first observed at the age of 9 months.

### mVD values in cortex and hippocampus of APP23 and WT mice

**Figure 3:**
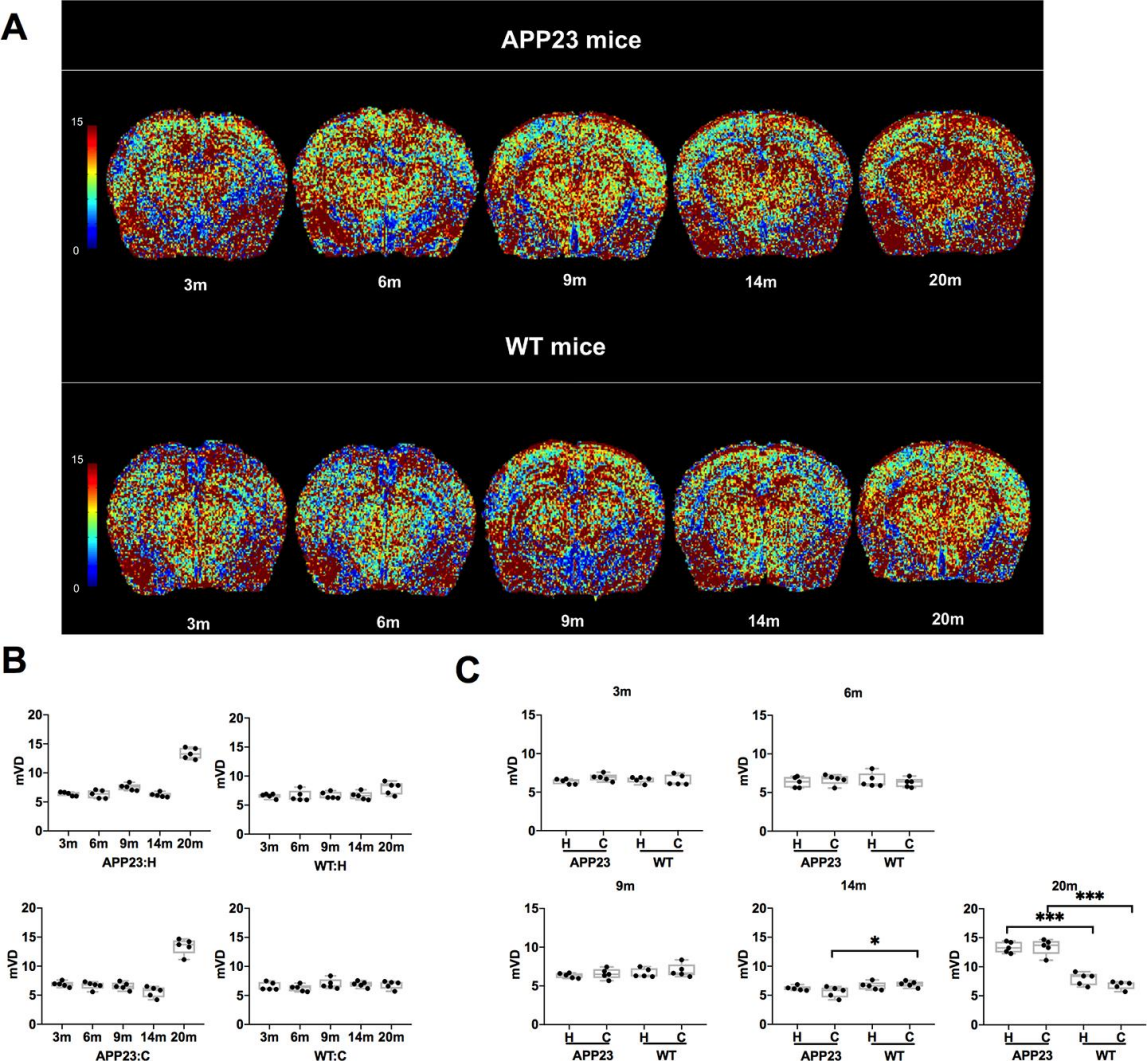
**mVD values in APP23 and WT mice of different ages.** (**A**) Colormap of mVD in APP23 and WT mice of different ages. (**B**) mVD values of cortex and hippocampus in APP23 and WT mice at different ages. (**C**) Comparison of mVD values in cortex and hippocampus between APP23 and WT mice at the same age. WT: wide type; C: cortex; H: hippocampus; mVD, mean vessel diameter in the voxel.

The mVD values of cortex and hippocampus in APP23 and WT mice of different ages are shown in Figure 3. The colormap of mVD in APP23 and WT mice at different ages was displayed in Figure 3A. As shown in Figure 3B, the mVD values of cortex and hippocampus in APP23 mice showed a slight decline from 3 to 14 months. However, at 20 months, the mVD values of cortex and hippocampus in APP23 mice significantly increased, indicating that the vascular diameter in APP23 mice was significantly enlarged at the late stage of aging. In contrast, in WT mice, there were no significant differences in the mVD values at different ages. Figure 3C illustrates the comparison of the mVD values in cortex and hippocampus between APP23 mice and WT mice at of the same ages. In the cortical area, the mVD value in 14-month old APP23 mice was significantly lower than in WT mice, but at 20 months, the mVD value of cortex in APP23 mice was significantly higher than in WT mice. In addition, in the hippocampal area, the mVD value in APP23 mice was significantly higher than in 20-month old WT mice. Thus, the mVD values indicate that significant differences between APP23 and WT mice are first observed in the cortex at the age of 14 months.

### VSI values in cortex and hippocampus of APP23 and WT mice

**Figure 4:**
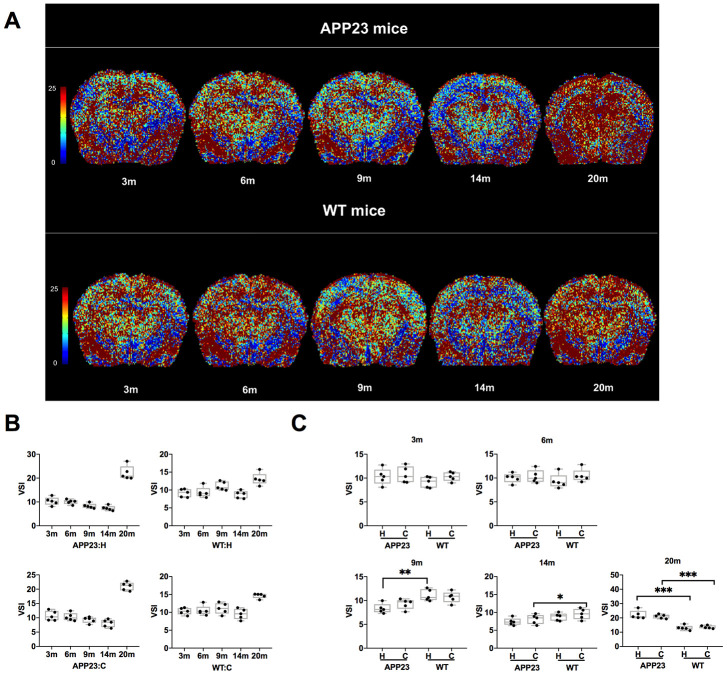
**VSI values in APP23 and WT mice of different ages.** (**A**) Colormap of VSI in APP23 and WT mice of different ages. (**B**) VSI values of cortex and hippocampus in APP23 and WT mice at different ages. (**C**) Comparison of VSI values in cortex and hippocampus between APP23 and WT mice at the same age. WT: wide type; C: cortex; H: hippocampus; VSI, vessel size index.

The VSI values in cortex and hippocampus of APP23 and WT mice at different ages are shown in Figure 4. The colormap of VSI in APP23 and WT mice at different ages was displayed in Figure 4A. As shown in Figure 4B, the VSI values in cortex and hippocampus of APP23 mice decreased gradually from 3 to 14 months. However, at 20 months, the VSI values in cortex and hippocampus in APP23 and WT mice significantly increased. The comparison of the VSI values in cortex and hippocampus between APP23 and WT mice of the same age is shown in Figure 4C. In 9 month-old APP23 mice, the hippocampal area VSI value was significantly lower than in WT mice, while, in the cortical area, the VSI value in 14-month old APP23 mice was significantly lower than in WT mice. In 20-month old APP23 mice, the VSI values of both cortex and hippocampus were significantly higher than WT mice. These results suggest that significant differences between APP23 and WT mice are first observed in the hippocampus at the age of 9 months, indicating that the hippocampus is the ‘sensitive region’ that reflects the early changes of microvessels in AD.

## DISCUSSION

AD is a progressive neurodegenerative disease with a high morbidity rate in the world [15, 16]. Recent studies have emphasized the important roles of vascular factors in AD [17–19]. However, the specific variation of microvessels at the early stages of AD have not been identified which may improve the early diagnosis of AD. In this study, we performed a non-invasive and quantitative assessment of vascular density and size in AD mouse model using vessel size imaging. Our results indicate that abnormal vascular density and morphology changes associated with AD can be identified as early as 9 months, and that hippocampus is the sensitive region that reflects the microvascular changes in AD.

### Vascular density changes in AD

By quantitative assessment of APP23 and WT mice of different ages, we found that the Density values of cortex and hippocampus decreased with age, especially at 20-month-old age. The age-associated decrease in Density values was also demonstrated by Fischer et al [20]. Furthermore, histopathology studies demonstrated that in older (20 months) APP23 mice, the exponentially increased Aβ plaques were widely distributed in cerebral cortex, hippocampus, capillary lumen, and other brain regions, resulting in an irreversible significant decrease of cerebral microvascular density [14, 21–23].

Our data demonstrated a transient increase in Density values in 14-month old APP23 mice which was significantly different from WT mice. We supposed that this transient increase in APP mice was compensatory. According to previous studies, microvascular pathological environment in APP23 mice induced the activation of macrophages and monocytes, which released the vascular endothelial growth factor (VEGF), basic fibroblast growth factor (bFGF), and platelet-derived growth factor (PDGF) [24–27]. Those growth factors maybe a potential cause of transient Density increase.

By comparing the cortical and hippocampal Density values in APP23 and WT mice of the same ages, a significant decrease was identified in hippocampus of APP23 mice at 9 months, indicating that hippocampus could be considered as the sensitive region in evaluating the vascular density of AD. This manifestation corresponded to the discovery of Aβ deposition in the hippocampus of APP23 mice at the age of 6-8 months, as demonstrated by Meyer's histopathological findings [28]. In addition, the cortex Density value in 14-month old APP23 mice was significantly higher than in WT mice, suggesting that the cortex could be considered as the specific region reflective of vascular Density changes in AD.

### Increased mVD values at the late stage of AD

mVD, a dimensionless ratio, represents the vascular size distribution and is estimated by the water diffusion coefficient and contrast agent concentration [29]. In this study, our results suggested that the vascular diameter of APP23 mice underwent a variation from slight constriction to obvious dilation with AD progression. Histopathologically, hypoxia and hypoperfusion in APP23 mice can lead to vascular contraction by the regulation of vascular contractile endothelin-1 (ET-1) and angiotensin II [18, 30]. Based on Magnetic resonance angiography (MRA) imaging methods, APP23 mice had abnormal hemodynamics in the circle of the Willis arteries at the age of 20 months [31]. Additionally, the severity of Aβ pathological deposition also contributes to the vascular diameter changes, including loss of vascular elasticity, degeneration of muscle cells and atrophy of vascular wall, especially in the late stage of AD [18]. By comparing the cortical and hippocampal mVD values in APP23 and WT mice of the same ages, significant differences between APP23 and WT mice were observed in the cortex at the age of 14 months, indicating that cortex could be considered as a region reflective of mVD changes in AD.

### Early vasoconstriction and late dilation of cerebrovascular microvessels in AD

VSI is the averaged microvessel size over the capillary population based on the weight of its volumetric fraction [11]. It is often used to detect the contraction and dilation of microvessels. In this study, the VSI values of cortex and hippocampus of APP23 mice decreased gradually from 3 to 14 months, but significantly increased at 20 months. The variation trend of VSI values in APP23 and WT mice was generally consistent with mVD values, and indicated that the variation of vascular size changed from slight constriction to obvious dilation with AD progression. However, the earlier abnormalities in vessel size and the more significant degree of late vasodilation detected by VSI indicated that VSI was more sensitive than mVD to monitor the vascular size. This finding is also supported by previous study of imaging and histological indicators demonstrating that VSI is most closely related to histopathological changes [11]. From the perspective of imaging processing, compared with mVD, VSI can avoid the strong dependence on the contrast agent concentration, and thus provides a more accurate and sensitive evaluation of the vascular size [29].

Comparing cortical and hippocampal VSI of APP23 and WT mice at the same age revealed that the hippocampal VSI value of 9-month old APP23 mice and the cortical VSI value of 14-month old APP23 mice were significantly different from those of WT mice. This finding verified that the hippocampus was a sensitive brain region involved in AD progression, and could be considered as a brain region reflective of vascular morphology changes during early stages of AD [32, 33].

### Limitations, future directions, and conclusions

This study focused on imaging of vascular abnormalities in APP23 transgenic mice. A better understanding of the vascular changes involved in AD will require a combination of histopathology, molecular biology, and imaging approaches in different AD models, which are our next focus. Additionally, we also attempted to combine the indicators of Density, mVD and VSI obtained by vessel size imaging to detect the altered pattern of microvascular density and morphology in human patients at the early stage of AD.

Together, our data show that the late stage (20-month) of AD progression in APP23 mice is associated with decreased Density of cerebral microvessels, increased vascular dilatation, and vascular diameter enlargement. In addition, our results demonstrate that the hippocampus is the most susceptible cerebral region in AD, and can be regarded as the critical area for monitoring the microvascular changes in AD. Our study provides novel imaging biomarkers for the early detection of AD, and for evaluating the therapeutic effects of vascular targeted therapy in AD.

## MATERIALS AND METHODS

### Animals

APP23 transgenic mice were provided by the Neurodegenerative Disorder Research Center, School of Life Sciences Material Science at Microscale National Laboratory, University of Science and Technology of China. APP23 mice contain a human amyloid precursor protein (*APP751*) cDNA with the Swedish double mutation at position 670/671 driven by the neuron-specific Thy-1 promoter [34], resulting in their seven-fold higher APP expression compared to the endogenous murine APP. APP23 mice suffer from dual impacts of vascular injury and Aβ deposition [35], which is characterized by vascular pathological changes and amyloid deposition in the blood vessels [22]. All mice were kept under a 12/12 h light/dark cycle, fed standard food, and had access to water ad libitum. Twenty-five heterozygote mice and twenty-five corresponding wild type (WT) mice were bred to reach a final study cohort of 50 mice. Both APP23 mice and WT mice were divided into five groups (n = 5 each), and analyzed at the age of 3, 6, 9, 14, and 20 months. No further inclusion or exclusion criteria were applied. The animal experiments were approved by the local Animal Ethics Committee and carried out in strict compliance with the National Institutes of Health Guidelines for Care and Use of Laboratory Animals.

### Magnetic resonance imaging (MRI)

All MRI experiments were performed on a horizontal 9.4 T/400 mm wide bore scanner (Agilent Technologies, Inc., Santa Clara, CA, USA), using a volume radiofrequency (RF) coil. Mice were anesthetized with isoflurane (3.5% induction, 1.0%-1.5% maintenance) in air/O_2_ (2:1) during scanning, and a catheter was placed in the tail vein. The respiratory rate and rectal temperature were monitored throughout the experiment with a physiologic monitoring unit (model 1030; SA Instruments, Inc., Stony Brook, NY, USA).

T_2_^*^ weighted imaging (T_2_^*^ WI) was obtained from the gradient-echo (GRE) MRI sequence, and the parameters were listed as follows: repetition time (TR) = 500 ms; echo time (TE) = 2.6, 6.52, 10.44, 14.36, and 18.28 ms; field of view (FOV) = 16 × 16 mm^2^; matrix size = 192 × 192; slice thickness = 1 mm (10 slices, gap = 0); 2 averages; and bandwidth (BW) = 100 kHz. T_2_ weighted imaging (T_2_ WI) was obtained from the spin-echo (SE) MRI sequence, and the parameters were listed as follows: TR = 3500 ms; eight evenly spaced spin-echoes = [9.45–75.58] ms; FOV = 16 × 16 mm^2^; matrix size = 192 × 192; slice thickness = 1 mm (10 slices, gap = 0); 2 averages; and BW = 50 kHz. Additionally, the apparent diffusion coefficient (ADC) map was obtained from a 12-directional diffusion-weighted imaging (DWI) sequence with b = 900 s/mm^2^ and a reference image (b ≈ 0 s/mm^2^) (TR = 3000 ms; TE = 27 ms; number of excitations (NEX) = 2; matrix = 192 × 192; FOV = 16 × 16 mm^2^; slice thickness = 1 mm (10 slices, gap = 0); BW = 50 kHz). Repeated gradient-echo and spin-echo MRI sequence scans were acquired after the injection of ultra-small superparamagnetic iron oxide (USPIO) contrast agent (Shanghai So-Fe Biomedical Co., Ltd.) via the tail vein over approximately 5 min. A dose of 0.02 mL per g of body weight was injected at a speed of 0.02 mL/s. The total MRI session lasted 1 h 20 min per animal.

### Data processing

### Region of interest

The ImageJ software was used to outline the brain region mask, excluding the skin and skull, on T_2_ weighted imaging (T_2_WI). Mask calibration was performed using the Matrix Laboratory (MATLAB) (Mathworks, Natwick, Massachusetts, USA). Two regions of interests (ROIs: cortex and hippocampus) were delineated on T_2_WI. Each ROI, delineated on T_2_WI, was transferred onto the T_2_^*^WI, ADC, ΔR_2_, $\Delta {\text{R}}_{2}^{*},$ mVD, density, and VSI maps [11].

ΔR_2_, the change in the transverse relaxation rate R_2_, was derived from the T_2_ maps pre- and post-USPIO injection as follows:

$$\Delta {\text{R}}_{2}=\frac{\text{1}}{{\text{T}}_{\text{2post}}}-\frac{\text{1}}{{\text{T}}_{\text{2pre}}}$$

$\Delta {\text{R}}_{2}^{*},$ the change in the relaxation rate ${\text{R}}_{2}^{*},$ was computed as follows:

$$\Delta {\text{R}}_{2}^{*}=\frac{1}{{\text{T}}_{\text{2post}}^{\text{*}}}-\;\frac{1}{{\text{T}}_{\text{2pre}}^{\text{*}}}$$

${\text{T}}_{2\text{pre}}^{\text{*}}$ and ${\text{T}}_{2\text{post}}^{\text{*}}$ were the pre- and post-injection relaxation times.

For each ROI, the ADC, $\Delta {\text{R}}_{2}^{*},$ and ΔR_2_ values were computed. The mVD was computed as follows:

$$\text{mVD}=\frac{{\text{ΔR}}_{\text{2}}^{\text{*}}}{{\text{ΔR}}_{\text{2}}}$$

Density was computed as follows:

$$\text{Density}=329\frac{{\left({\text{ΔR}}_{\text{2}}\right)}^{\text{3}}}{{\left({\text{ΔR}}_{\text{2}}^{\text{*}}\right)}^{\text{2}}}$$

The VSI was computed as follows:

$$\text{VSI}=0.424{\left(\frac{\text{ADC}}{{\text{γΔχB}}_{0}}\right)}^{1/2}{\left(\frac{{\text{ΔR}}_{2}^{\text{*}}}{{\text{ΔR}}_{2}}\right)}^{3/2}$$

The ADC was computed as the mean of the ADCs observed in the 12 directions of the gradient system. Furthermore, B_0_ represented the static magnetic field with the value of 9.4 T; γ represented the gyromagnetic ratio of the protons, and the gyromagnetic ratio of hydrogen protons was 42.58 MHz/T; Δχ was considered equal to 0.57 ppm. The unanalyzed voxels (i.e., non-converging fit and values outside the range of validity (ADC > 3,000 μm^2^/s; ΔR_2_ ≤ 0; $\Delta {\text{R}}_{2}^{*}\leq 0$) were excluded from further consideration.

### Statistical analysis

All results were expressed as the mean ± standard deviation. Paired Student’s t-tests were used for the comparisons between the cortex and hippocampus, along with APP23 and WT mice. The longitudinal assessment of microvascular characteristics, including the mVD, density, and VSI, were evaluated with Analysis of Variance (ANOVA).
